# Adenoviral intramyocardial VEGF-D^ΔNΔC^ gene transfer increases myocardial perfusion reserve in refractory angina patients: a phase I/IIa study with 1-year follow-up

**DOI:** 10.1093/eurheartj/ehx352

**Published:** 2017-07-31

**Authors:** Juha Hartikainen, Iiro Hassinen, Antti Hedman, Antti Kivelä, Antti Saraste, Juhani Knuuti, Minna Husso, Hanna Mussalo, Marja Hedman, Tuomas T. Rissanen, Pyry Toivanen, Tommi Heikura, Joseph L. Witztum, Sotirios Tsimikas, Seppo Ylä-Herttuala

**Affiliations:** 1 Heart Center, Kuopio University Hospital, Kuopio 70029, Finland; 2 Institute of Clinical Medicine, University of Eastern Finland, Kuopio 70211, Finland; 3 Turku PET Centre, Turku University Hospital, Turku 20521, Finland; 4 Center of Diagnostic Imaging, Kuopio University Hospital, Kuopio 70029, Finland; 5 Heart Center, Central Hospital of North Karelia, Joensuu 80210, Finland; 6 A.I. Virtanen Institute, University of Eastern Finland, Kuopio 70211, Finland; 7 University of California San Diego, La Jolla, CA 92093, USA; 8 Gene Therapy Unit, Kuopio University Hospital, Kuopio 70029, Finland

**Keywords:** Gene therapy, Angiogenesis, Lymphangiogenesis, Therapeutic angiogenesis, PET, Safety

## Abstract

**Aims:**

We evaluated for the first time the effects of angiogenic and lymphangiogenic AdVEGF-D^ΔNΔC^ gene therapy in patients with refractory angina.

**Methods and results:**

Thirty patients were randomized to AdVEGF-D^ΔNΔC^ (AdVEGF-D) or placebo (control) groups. Electromechanical NOGA mapping and radiowater PET were used to identify hibernating viable myocardium where treatment was targeted. Safety, severity of symptoms, quality of life, lipoprotein(a) [Lp(a)] and routine clinical chemistry were measured. Myocardial perfusion reserve (MPR) was assessed with radiowater PET at baseline and after 3- and 12-months follow-up. Treatment was well tolerated. Myocardial perfusion reserve increased significantly in the treated area in the AdVEGF-D group compared with baseline (1.00 ± 0.36) at 3 months (1.31 ± 0.46, *P* = 0.045) and 12 months (1.44 ± 0.48, *P* = 0.009) whereas MPR in the reference area tended to decrease (2.05 ± 0.69, 1.76 ± 0.62, and 1.87 ± 0.69; baseline, 3 and 12 months, respectively, *P* = 0.551). Myocardial perfusion reserve in the control group showed no significant change from baseline to 3 and 12 months (1.26 ± 0.37, 1.57 ± 0.55, and 1.48 ± 0.48; respectively, *P* = 0.690). No major changes were found in clinical chemistry but anti-adenovirus antibodies increased in 54% of the treated patients compared with baseline. AdVEGF-D patients in the highest Lp(a) tertile at baseline showed the best response to therapy (MPR 0.94 ± 0.32 and 1.76 ± 0.41 baseline and 12 months, respectively, *P* = 0.023).

**Conclusion:**

AdVEGF-D^ΔNΔC^ gene therapy was safe, feasible, and well tolerated. Myocardial perfusion increased at 1 year in the treated areas with impaired MPR at baseline. Plasma Lp(a) may be a potential biomarker to identify patients that may have the greatest benefit with this therapy.

## Introduction

Angina pectoris is the most common symptom of coronary artery disease (CAD). In spite of improved medical and revascularization therapies, 5–10% of patients undergoing coronary angiography have refractory angina (RA), i.e. they are severely symptomatic while on optimal medical therapy and prior revascularization and not amenable to further revascularization procedures.^1^^,^^2^ In the EU and USA, there are more than 200 000 new RA patients/year.^2^ Thus, an unmet clinical need exists for new therapies for this group of patients.^1^^,^^2^

Some patients with CAD develop collateral arteries, which can rescue ischaemic myocardium in spite of significant occlusions in coronary arteries and alleviate ischaemic symptoms. Therapeutic vascular growth stimulates this natural process and offers a potential new treatment for RA.^3–6^ However, most previous cardiovascular proangiogenic trials have been unsuccessful.^7–11^ This is likely due to (i) poor gene transfer efficiency in the myocardium, (ii) tested growth factors may not have been the most optimal ones, and (iii) inability to target therapy into ischaemic, but viable myocardium.^3^^,^^4^

To address these challenges, we used PET perfusion imaging and an electromechanical catheter system for gene transfer to identify ischaemic, hibernating myocardium with the lowest perfusion reserve for the targeted therapy. For the first time, we also used VEGF-D^ΔNΔC^, a new member of the VEGF family that stimulates both angiogenesis and lymphangiogenesis.^12^^,^^13^ In addition, because Lp(a) is associated with pro-atherogenic, pro-inflammatory, and pro-thrombotic effects, elevated plasma levels were tested as a potential new biomarker to identify patients who might benefit from the induced therapeutic vascular growth.^14^

## Methods

KAT301 is a randomized, blinded, controlled phase I/IIa trial which assessed the safety and feasibility (primary end points) of targeted intramyocardial gene therapy in RA patients using adenoviruses (Ad) expressing human VEGF-D^ΔNΔC^. In addition, we assessed effects on myocardial perfusion reserve (MPR), improvement in symptoms [Canadian Cardiac Society Class (CCS Class)], and quality of life (QoL) at 3 and 12 months (secondary end points). Institutional review board and Finnish authorities approved the protocol. Patients gave written informed consent. Trial design is presented in Supplementary material online. Trial was registered at Clinical Trials Gov NCT01002430 and EudraCT 003295-22.

### Patients and endocardial mapping

**Figure 1:**
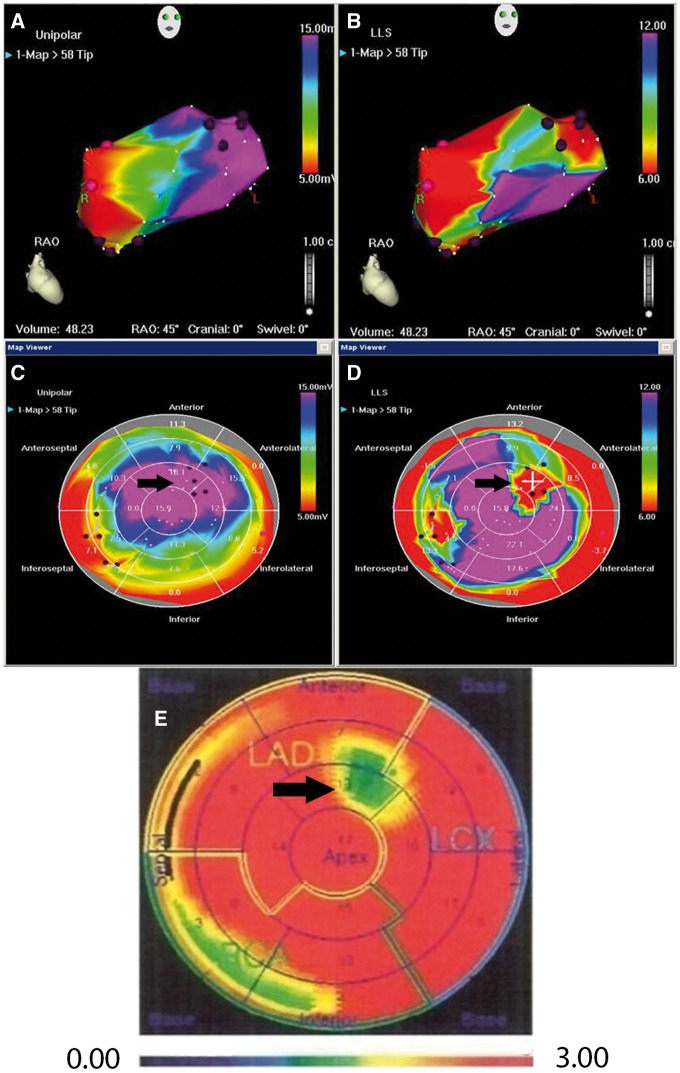
NOGA (*A*–*D*) and PET radiowater images (*E*). Panels *A*–*B* are three-dimensional NOGA maps of the left ventricle and Panels *C*–*D* represent A-B converted to two-dimensional bull’s eye views. Panels *A* and *C* show myocardial viability (unipolar voltage maps). Purple colour in *A* and *C* indicates normal viability (black arrow). Panels *B* and *D* show myocardial contractility (local linear shortening maps). Red colour indicates areas with reduced contractility (black arrow). Panel *E* shows PET data in bull’s eye view. Black arrow indicates an area with reduced blood flow (green) which is the same area as seen in Panel *C* with normal viability and in Panel *D* with poor contractility. This area was treated with gene therapy. Black dots indicate injection sites in *C*–*D*. Colour scale in NOGA maps: purple (viable myocardium or normal contractility)-blue/green/yellow (reduced/low viability or contractility)-red (non-viable myocardium or poor contractility); Colour scale in PET map: red (best perfusion)-yellow/green (reduced/low perfusion)-blue (poor/no perfusion).

Thirty patients with severe RA were randomized 4:1 to VEGF-D^ΔNΔC^ therapy (AdVEGF-D group) and placebo (controls) in blocks of five patients. After transseptal puncture, an 8.5Fr introducer catheter (AgilisTM NxT St Jude Medical, USA) and an electroanatomical mapping and injection catheter (NOGA©, Johnson & Johnson, USA) were introduced into left ventricle. To select optimal sites for gene injections, the left ventricle was mapped to detect areas of viable myocardium with reduced contraction (*Figure 1*). Coronary angiography and baseline radiowater PET imaging^15^ were used to confirm viable myocardial segments with impaired MPR (*Figure 1*).

### Gene transfer

Following mapping, the randomization code was opened in hospital pharmacy. NOGA© catheters were used to inject AdVEGF-D^ΔNΔC^ to 10 different sites (200 µl each) in the target myocardium. The control group underwent the same mapping procedure. Ten 200 μl injections of 0.9% NaCl were given into the selected sites with the needle withdrawn. Only the operator and hospital pharmacy were open to the randomization code. Other personnel and patients were blinded throughout the study.

### Adenoviral vector and VEGF-D^ΔNΔC^

Replication-deficient E1-E3-deleted serotype 5 adenoviruses were produced in 293 cells by FinVector Therapies Oy (Kuopio, Finland).^16^ AdVEGF-D^ΔNΔC^ is an angiogenic and lymphangiogenic growth factor which contains a VEGF homology domain but lacks the N- and C-terminal propeptides.^17^^,^^18^ Details of the biological effects and signalling of AdVEGF-D^ΔNΔC^ have been described.^12^^,^^17^^,^^18^

### Perfusion imaging

Quantitative myocardial perfusion was determined with PET at baseline and at 3 and 12 months.^15^ A dynamic PET scan was performed (^15^O-H_2_O; 900–1100 MBq) at rest and during adenosine stress. Regional myocardial blood flow (MBF) was measured in 17 segments as an average of three repeated analyses (Carimas software 2.5; www.turkupetcentre.net/carimasturku) blinded to the treatment and clinical data.^19^ Myocardial perfusion reserve was calculated for each segment as the ratio of MBF during adenosine stress and at rest. Two areas of interest were defined: (i) low MPR defined as the myocardial area with the lowest MPR and (ii) reference MPR as the myocardial segment with the highest MPR at baseline.^19^

### Quality of life and angina symptoms

Quality of life was assessed with a standardized 15 dimensions (15D) questionnaire at baseline and 3 months. The single index (15D score) on a 0–1 scale represents overall QoL.^20^ The maximum score is 1 (no problems on any dimensions) and the minimum is 0. A chance of ≥0.015 in the 15D score is clinically significant.^21^ CCS Class was evaluated at baseline and during the follow-up using standard methods.

### Statistical analysis

Repeated measurements were analysed with linear mixed effect model and *posthoc* analyses were performed by the least significance method. Mann–Whitney *U* test was used to compare differences between the groups for continuous variables. Fisher’s exact test was used to calculate dichotomous variables. Results are expressed as means ± standard deviation for continuous variables, and as absolute and relative frequencies for categorical variables. Odds ratios and 95% confidence intervals were used to evaluate associations. Results were considered significant at *P* < 0.05 (SPSS Statistics version 21.0).

## Results

### Baseline characteristics and safety

The study groups were well balanced for baseline characteristics (*Table 1*). No significant differences were found between the AdVEGF-D and the control groups in early procedure-related (<14 days) or long-term (<360 days) adverse events (*Table 2*). No significant difference was found in MACE between the groups (*Table 2*).

**Table 1:** Baseline characteristics

	Control	AdVEGF-D	*P*-value
	*n* = 6	*n* = 24	
Demographics			
Sex (male/female)	5/1 (83%/17%)	23/1 (96%/4%)	0.67
Age (years)	70 ± 6	71 ± 6	0.40
CCS-class	2.67 ± 0.52	2.83 ± 0.38	0.56
Medical history			
Previous MI	4 (67)	17 (71)	0.60
Previous CABG	6 (100)	23 (96)	0.80
Previous PCI	3 (50)	15 (63)	0.46
Family history of CAD	5 (83)	19 (79)	0.66
Hypertension	6 (100)	22 (92)	0.63
Hypercholesterolaemia	6 (100)	23 (96)	0.80
Smoker (current/ex)	0/4 (0/67)	0/17 (0/71)	0.90
Diabetes	3 (50)	12 (50)	1.00
Drug therapy			
Aspirin	5 (83)	22 (92)	0.51
Clopidogrel	3 (50)	12 (50)	1.00
Warfarin	2 (33)	8 (33)	1.00
B-blockers	6 (100)	24 (100)	1.00
ACEI/ARB	6 (100)	21 (88)	0.49
Statins	5 (83)	24 (100)	0.20
Long-acting nitrates	5 (83)	23 (96)	0.37

Mean ± SD or *n* (%).

ACEI, angiotensin converting enzyme inhibitors; ARB, angiotensin receptor blockers; CAD, coronary artery disease; CABG, coronary artery bypass grafting; CCS, Canadian Cardiovascular Society; MI, myocardial infarction; PCI, percutaneous coronary intervention; SD, standard deviation.

**Table 2:** Adverse events

	Control	AdVEGF-D	OR (95% CI)	*P-*value
	*n* = 6	*n* = 24		
**Major complications**					
Death	0 (0)	3 (13)	2.12 (0.10–46.53)	0.50
During 14-day follow-up	0 (0)	1 (4)	0.83 (0.03–22.87)	0.80
ACS or MI	1 (17)	3 (13)	0.65 (0.06–7.64)	0.61
During 14-day follow-up	0 (0)	0 (0)	0.27 (0.00–14.69)	1.00
Stroke	0 (0)	2 (8)	1.47 (0.06–34.50)	0.63
During 14-day follow-up	0 (0)	1 (4)	0.83 (0.03–22.87)	0.80
MACE	1 (17)	8 (33)	2.50 (0.25–25.25)	0.40
**Other complications**					
Minor bleeding	2 (33)	9 (38)	1.20 (0.18, 7.93)	0.62
Procedural complications	1 (17)	1 (4)	0.22 (0.01, 4.09)	0.37
New atrial fibrillation	0 (0)	1 (4)	0.83 (0.03, 22.87)	0.80
Pericardial effusion	1 (17)	7 (29)	2.06 (0.20, 20.96)	0.48
During 14-day follow-up	0 (0)	5 (21)	3.67 (0.18–75.75)	0.30

Values are *n* (%). MACE = combined end point of death, ACS, MI, or stroke.

ACS, acute coronary syndrome; CI, confidence interval; MACE, major adverse cardiovascular event; MI, myocardial infarction; OR, odds ratio.

### Laboratory analyses and haemodynamics

**Figure 2:**
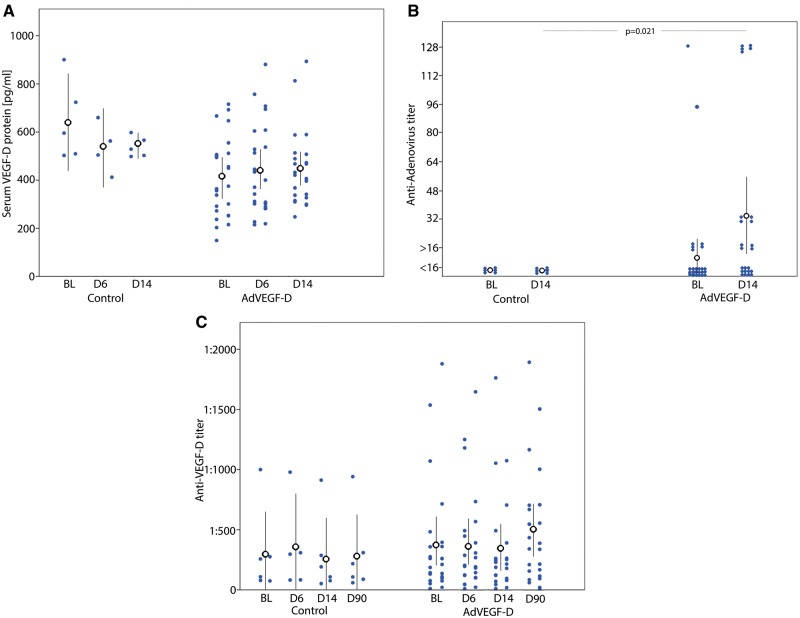
Serum VEGF-D protein levels (*A*), anti-adenoviral titers (*B*), and anti-VEGF-D titers (*C*) at baseline (BL) and 6 (D6), 14 (D14) and 90 (D90) days after gene transfer in controls (control) and AdVEGF-D^ΔNΔC^ treated patients (AdVEGF-D). Black dots and lines represent mean ± standard deviation. Blue dots represent values of individual patients.

Plasma Tnt increased in both AdVEGF-D and control groups on the first post-operative day (*P* = 0.001 and 0.004, respectively). Small decreases were also detected in plasma haemoglobin and platelet concentrations on Day 1 in the AdVEGF-D group (*P* = 0.011 and 0.001, respectively). C-reactive protein increased in the AdVEGF-D group on Day 6 (*P* < 0.002) (*Table 3*). No changes were found in plasma VEGF-D protein concentration (*Figure 2A*) whereas elevated anti-adenovirus antibody titer (≥16) was found in 54% of the AdVEGF-D patients 14 days after the gene transfer (*P* = 0.021) but in none of the controls (*Figure 2B*). An elevated anti-VEGF-D antibody titer (increase ≥four-fold) was detected in two AdVEGF-D patients at 3 months but in none of the controls (*Figure 2C*).

**Table 3:** Follow-up measurements

	Baseline		1 day		6 days		14 days		3 months		12 months		*P*-value
	Control	AdVEGF-D	Control	AdVEGF-D	Control	AdVEGF-D	Control	AdVEGF-D	Control	AdVEGF-D	Control	AdVEGF-D	
	*n* = 6		*n* = 6		*n* = 6				*n* = 6		*n* = 6		
												*n* = 24	
										*n* = 24			
							*n* = 6	*n* = 24					
				*n* = 24		*n* = 24							
		*n* = 24											
B-Hb (g/L)	143 ± 15	141 ± 16	138 ± 14	136 ± 16	137 ± 19	138 ± 14	135 ± 18	137 ± 15	142 ± 17	141 ± 15	145 ± 18	142 ± 18	0.011
B-Leuc (×10^9^/L)	8.4 ± 2.6	6.6 ± 1.4	7.8 ± 1.7	7.0 ± 1.5	7.2 ± 1.5	7.6 ± 4.0	6.9 ± 1.2	6.1 ± 1.1	7.9 ± 2.1	6.9 ± 1.2	8.6 ± 2.8	7.0 ± 1.2	0.569
B-Thromb (×10^9^/L)	274 ± 70	214 ± 63	247 ± 53	186 ± 61	289 ± 38	218 ± 75	269 ± 29	229 ± 72	247 ± 61	201 ± 59	261 ± 58	205 ± 86	0.001
P-Alt (U/L)	34 ± 17	32 ± 28	NA	NA	39 ± 20	30 ± 17	31 ± 13	30 ± 15	32 ± 24	29 ± 15	34 ± 16	26 ± 11	0.277
P-CRP (mg/l)	4 ± 2	4 ± 2	6 ± 3	9 ± 8	5 ± 3	13 ± 29	3 ± 0	4 ± 3	4 ± 2	3 ± 0	5 ± 3	3 ± 0	0.022
P-Tnt (ng/L)	10 ± 4	14 ± 6	95 ± 56	93 ± 39	12 ± 2	21 ± 17	11 ± 5	16 ± 8	14 ± 4	16 ± 9	14 ± 6	14 ± 9	0.001
Systolic BP (mmHg)	158 ± 16	141 ± 20	134 ± 25	131 ± 16	NA	NA	NA	NA	151 ± 21	141 ± 22	148 ± 21	140 ± 20	0.886
Diastolic BP (mmHg)	88 ± 10	80 ± 13	74 ± 12	71 ± 15	NA	NA	NA	NA	83 ± 11	76 ± 13	82 ± 21	76 ± 13	0.018
CCS class	2.67 ± 0.52	2.83 ± 0.38	NA	NA	NA	NA	NA	NA	2.17 ± 0.75	2.43 ± 0.59	2.00 ± 0.71	2.11 ± 0.47	0.001

Mean ± SD. *P*-value for interaction time x group. *P*-values for all comparisons in Supplementary material online, *Table S1*.

TnT, Troponine-T, BNP, N-terminal pro-brain natriuretic peptide; CCS, Canadian Cardiovascular Society class.

### Myocardial perfusion

**Figure 3:**
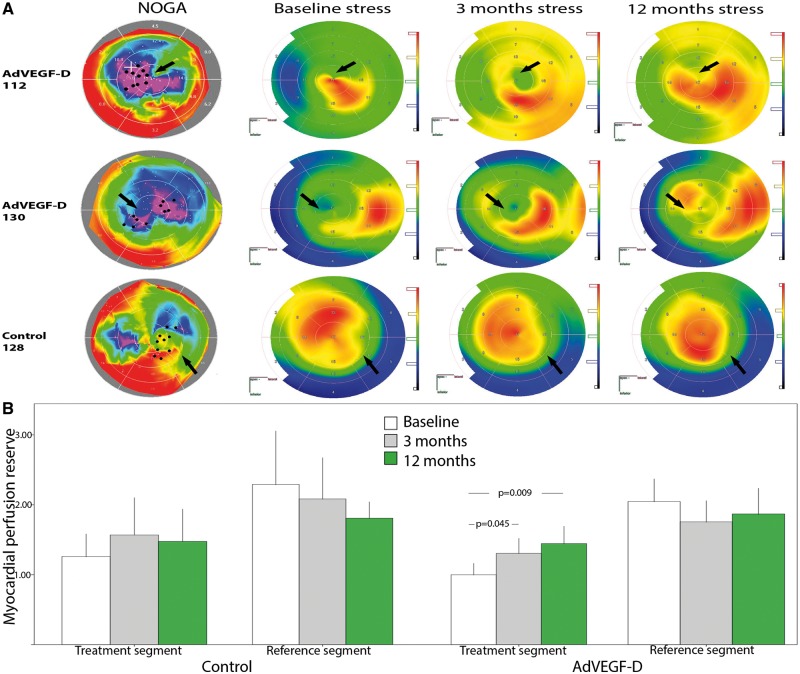
(*A*) Representative images of combined NOGA and stress PET radiowater images of two AdVEGF-D treated patients and one control patient. Black dots and arrows indicate sites for gene injections in viable but poorly perfused myocardium. Myocardial blood flow improved in the AdVEGF-D patients visualized as increases in red colour during the follow-up. Perfusion did not increase in the control patient. (*B*) Myocardial perfusion reserve in the treated and reference segments of the control and AdVEGF-D^ΔNΔC^-treated patients. Colour scales in NOGA and PET maps as in *Figure 1*. Values are mean ± standard deviation.

In the AdVEGF-D group, MPR of the treated area increased from 1.00 ± 0.36 at baseline to 1.31 ± 0.46 at 3 months (*P* = 0.045) and to 1.44 ± 0.48 at 12 months (*P* = 0.009) (*Figure 3AandB*). Myocardial perfusion reserve of the reference area (myocardium with the highest MPR at baseline) showed no significant change (2.05 ± 0.69, 1.76 ± 0.62, and 1.87 ± 0.69 at baseline, 3, and 12 months, respectively, *P* = 0.551). On the contrary, it tended to decrease by 10.7% and 8.8%, respectively (*Figure 3B*). Myocardial perfusion reserve in the control group showed no significant change from baseline to 3 and 12 months (1.26 ± 0.37, 1.57 ± 0.55, and 1.48 ± 0.48; respectively, *P* = 0.690) (*Figure 3B*).

No significant changes were found in the MPR of the reference area at baseline (2.29 ± 0.94), 3 months (2.08 ± 0.61, *P* = 0.550), or 12 months (1.81 ± 0.25, *P* = 0.647). In an exploratory analysis, AdVEGF-D patients belonging to the highest baseline Lp(a) tertile had the best response to the therapy (MPR 0.94 ± 0.32 and 1.76 ± 0.41, baseline and 12 months, respectively, *P* = 0.023, *Table 4*).

**Table 4:** Myocardial perfusion reserve and Lp(a) levels of the AdVEGF-D^dNdC^ treated patients

	**Baseline**			**3 months**			**12 months**			***P-*value**
Lp (a) tertiles	3rd	2nd	1st	3rd	2nd	1st	3rd	2nd	1st	
	*n* = 5	*n* = 5	*n* = 5	*n* = 5	*n* = 5	*n* = 4	*n* = 5	*n* = 5	*n* = 5	
Lp(a) mg/dL	52.7 ± 13.3	11.8 ± 6.4	3.8 ± 2.0	53.9 ± 20.5	11.0 ± 5.0	3.2 ± 1.3	59.1 ± 19.6	13.8 ± 4.1	3.3 ± 0.7	0.023
Treated area MPR	0.94 ± 0.32	1.16 ± 0.45	1.03 ± 0.34	1.43 ± 0.45	1.39 ± 0.57	1.17 ± 0.38	1.76 ± 0.41	1.42 ± 0.53	1.22 ± 0.	0.089

Mean ± SD. Tertiles refer to Lp(a) at baseline. *P*-value for interaction time x tertiles. *P*-values for all comparisons in Supplementary material online, *Table S2*.

MPR, myocardial perfusion reserve; SD, standard deviation; Lp(a), lipoprotein(a).

### Angina pectoris

There was a significant improvement in angina pectoris symptoms (CCS class) in the AdVEGF-D group at 12 months (2.83 ± 0.38 vs. 2.11 ± 0.47, *P* = 0.001) (*Table 3*). CCS class tended to improve also in the controls but it did not reach statistical significance (2.67 ± 0.52 vs. 2.00 ± 0.71, *P* = 0.279).

### Quality of life

In the AdVEGF-D group, the 15D analysis score increased from 0.787 ± 0.108 at baseline to 0.803 ± 0.101 at 3 months. Thus, a clinically meaningful change (change  ≥ +0.015) in the mean 15D score was observed at 3 months (+0.016). 15D scores in the controls (baseline 0.788 ± 0.129 and 3 months 0.790 ± 0.123) showed no statistical or clinically meaningful changes.

## Discussion

Refractory angina refers to patients with CAD who suffer from chest pain and disability despite optimal medical and revascularization therapy and who are not eligible for additional coronary interventions.^1^^,^^2^ Therapeutic vascular growth (i.e. stimulation of both angiogenesis and lymphangiogenesis) is a new concept for the treatment of RA.^4^ The main finding in our study was that intramyocardial AdVEGF-D^ΔNΔC^ gene therapy was safe, feasible, and well tolerated. However, it is an invasive procedure and resulted in transient increases in plasma troponin, which were most likely due to the transseptal approach, mapping of the left ventricle, and intramyocardial injections. An increase in anti-adenovirus antibodies in 54% of the treated patients was an expected finding^6^ with no clinical consequences. However, if readministration of the AdVEGF-D^ΔNΔC^ would be required, the increased antibody levels might reduce the efficacy of the second administration. A modest decrease in diastolic blood pressure in the AdVEGF-D group on the first post-operative day was likely caused by an increased NO production induced by VEGF-D^ΔNΔC^.^12^ A potential impact of elevated Lp(a) was also noted in the response of the RA patients to this therapy, with the most benefit in patients with the highest Lp(a) levels. This is consistent with a recent report that 50% of patients with RA have elevated Lp(a) (>50 mg/dL), and in whom Lp(a) lowering achieved by lipid apheresis was associated with objective evidence of myocardial blood flow improvement by MRI and significant relief of RA symptoms.^22^

There were no significant differences between the AdVEGF-D and control groups with respect to major complications. However, because of the small number of patients, these results should be interpreted cautiously. Three AdVEGF-D patients died during the follow-up. One death was due to myocardial infarction (MI) and two were sudden deaths. Patients suffering from sudden death underwent 24 h electrocardiogram recordings without any major arrhythmias (data not shown). In the AdVEGF-D group, three patients and one patient in the control group developed acute coronary syndrome (ACS) or MI during the follow-up. Coronary angiogram was available from three of these patients and in all of them a new stenosis in vein graft was responsible for the event. Thus, they were unlikely to be related to gene therapy. Mild pericardial effusion was found in 29% of the AdVEGF-D patients and 17% of the controls. VEGF-D^dNdC^ is known to increase not only perfusion but also vascular permeability, albeit much less than VEGF-A.^12^^,^^18^^,^^23^ Thus, it is likely that pericardial effusion in the gene-treated patients mirrors the biological effect of VEGF-D^ΔNΔC^. However, in all cases the mild effusions recovered spontaneously without sequelae and required no therapy.

Although the primary target of our study was the safety and feasibility it is of interest that myocardial perfusion increased in the treated myocardial segments at 3 and 12 months. To our knowledge, this is the first time that treatment aimed to cause vascular growth shows an objective long-term improvement in myocardial perfusion in the treated areas. Myocardial perfusion in the reference segments of the same patients as well as in the control group tended to decrease, which suggests progression of the underlying CAD.

In comparison to earlier studies,^7–11^ an important advancement in this study was that we selected VEGF-D^ΔNΔC^ as the therapeutic gene. It has several advantages over the previously used growth factors, such as VEGF-A: (i) AdVEGF-D^ΔNΔC^ has slower but more long-lasting signalling kinetics than VEGF-A through VEGF Receptor-2 thus providing a more sustained angiogenic stimulus^12^; (ii) It does not bind to matrix proteoglycans and diffuses better than VEGF-A in the transduced tissues^12^^,^^13^; (iii) AdVEGF-D^ΔNΔC^ binds to Neuropilin-1 and -2 which strengthens angiogenic responses^12^; (iv) AdVEGF-D^ΔNΔC^ stimulates lymphatic vessel growth via VEGF Receptor-3 which improves fluid drainage from the treated myocardium^23^; (v) AdVEGF-D^ΔNΔC^ induces less changes in vascular permeability than VEGF-A and reduces the risk of pericardial fluid accumulation^23^; (vi) AdVEGF-D^ΔNΔC^ does not bind to VEGF Receptor-1 on monocytes and therefore does not directly stimulate inflammatory mechanisms as compared with VEGF-A^24^; and (vii) Unlike some other VEGFs, AdVEGF-D^ΔNΔC^ does not induce ventricular arrhythmias in the compromised heart muscle.^24^

Another advancement in our study was that we used combined electromechanical mapping and PET perfusion imaging for the selection of the treatment area (*Figure 1*) as well as for the assessment of changes in MBF during the follow-up (*Figure 3*). SPECT has been used in previous gene therapy trials^7^^,^^10^^,^^11^ but it can only be analysed semiquantitatively using differences in relative counts between rest and stress as compared with areas with normal perfusion.^25^ Therefore, SPECT has limited sensitivity for assessing regional perfusion in patients with severe CAD and cannot be used to assess quantitative changes in MPR. PET permits the measurement of absolute myocardial blood flow and MPR from rest to stress states, providing an objective, validated surrogate end point.^26^ Reproducibility of quantitative MBF and MPR measurements are sufficient for the detection of significant changes (coefficient of variation 15%).^26^

Lipoprotein(a) is a risk factor for MI, stroke, and peripheral arterial disease.^14^^,^^27^ Since Lp(a) has strong prothrombotic and antiangiogenic activity, we hypothesized that Lp(a) could be used as a biomarker to identify patients who might benefit from gene therapy. AdVEGF-D patients in the highest baseline Lp(a) tertile had a significant improvement in MPR as compared with those in the lowest Lp(a) tertile at 12 months. If confirmed, Lp(a) could be used to identify patients who might optimally benefit from AdVEGF-D gene therapy. Lp(a) and oxidized phospholipids they carry are abundantly present in vulnerable plaques, in debris from distal protection devices and in chronic total occlusions, plaque phenotypes that often lead to MI and RA.^27^^,^^28^ Lipoprotein(a) is also highly pro-inflammatory and mediates secretion of cytokines from monocytes that promotes arterial inflammation.

We found a significant improvement in angina pectoris symptoms (CCS class) and a clinically important improvement in QoL (change  ≥ +0.015 in the mean 15D score) in the AdVEGF-D patients. A similar trend in symptoms was also found in the controls. As the controls underwent the same intracardiac mapping procedure, we cannot exclude a possible placebo effect, which has been found in previous gene therapy trials.^3^^,^^4^ Establishing clinical significance of the CCS and QoL findings requires further studies.

Randomized controls were catheterized and mapped exactly in the same way as the AdVEGF-D patients but 0.9% NaCl placebo solution was injected without pushing the needle out from the catheter. Only the operator and hospital pharmacy providing AdVEGF-D or placebo solutions were aware of the treatment. Other study personnel responsible for the patient care, data analysis and follow-up were blinded to the treatment. To maintain blinding, all antibody and VEGF-D^ΔNΔC^ measurements were performed only at the end of the study. Other options for the placebo administration could have been the use of an empty adenovirus and/or intramyocardial injections of placebo. However, these were considered unethical by the institutional review board.

Limitations of this study include the small number of patients, which precludes any firm conclusions about safety and efficacy. A lack of statistical significance does not necessarily confirm the lack of difference. Particularly this is true for clinical end points. Thus, potential rare complications of the AdVEGF-D^ΔNΔC^ gene therapy procedure may not have been detected.

In conclusion, NOGA catheter-mediated intramyocardial delivery of AdVEGF-D^ΔNΔC^ was safe and well tolerated and may offer a new option for the treatment of RA. To our knowledge, this is the first study demonstrating a significant improvement in quantitative myocardial blood flow after local gene therapy in the treated areas with impaired perfusion reserve. Elevated plasma Lp(a) may be used as a biomarker to identify patients who could benefit from the AdVEGF-D^ΔNΔC^ gene therapy. Phase IIb/III trials are needed to confirm the safety and efficacy of gene therapy in RA patients.

## Supplementary material

Supplementary material is available at *European Heart Journal* online.

## Supplementary Material

Supplementary Data

Supplementary Table 1

Supplementary Table 2
