# Corrigendum to: Adenoviral intramyocardial VEGF-D^ΔNΔC^ gene transfer increases myocardial perfusion reserve in refractory angina patients: a phase I/IIa study with 1-year follow-up

**DOI:** 10.1093/eurheartj/ehx576

**Published:** 2017-10-04

[Eur Heart J (2017); 38(33): 2547–2555]

The authors of the above article wish to inform readers that the amount given in Table 4, column ‘*P*-value’, row ‘Lp(a) mg/dL’ has been corrected from 0.203 to 0.023 via a post-publication correction.

The article has now been corrected online.

