# Supplementary material for: Adenoviral intramyocardial VEGF-DΔNΔC gene transfer increases myocardial perfusion reserve in refractory angina patients: a phase I/IIa study with 1-year follow-up
Source: Eur Heart J. 2017 Jul 31;38(33):2547–55. doi: 10.1093/eurheartj/ehx352 (PMC5837555; doi:10.1093/eurheartj/ehx352)
Supplement: Supplementary Data [file supplement_29.3_mr_ehx352.docx]

**Supplement material**

**Material and Methods**

**Patients and randomization**

KAT301 is a randomized, blinded, controlled phase I/IIa trial conducted at Kuopio and Turku University Hospitals (Finland). Thirty patients with severe RA were recruited and randomized 4:1 to VEGF-D^∆N∆C^ therapy (AdVEGF-D group) and placebo (control group) in blocks of five patients. An escalating dose of intramyocardial AdVEGF-D^∆N∆C^ was used for the first fifteen patients. Patients 1-5 were treated with 1x10^9^vpu AdVEGF-D^∆N∆C^ or placebo (0.9 % NaCl), patients 6-10 with 1x10^10^vpu AdVEGF-D^∆N∆C^ or placebo and patients 11-15 with 1x10^11^vpu AdVEGF-D^∆N∆C^ or placebo. Based on a protocol-based interim safety analysis of the first 15 patients, the highest dose of 1x10^11^vpu AdVEGF-D^∆N∆C^ or placebo was used for patients 16-30.

Inclusion criteria were as follows: signed informed consent; age between 30 to 80 years; severe angina pectoris (CCS II-III) despite of maximal medication; angina pectoris or ischemic ST-depression (≥1mm) in the exercise test; significant stenoses in coronary angiography; contraindication to PCI or CABG (diffuse or distal stenosis, chronic total occlusion not feasible for PCI, vessels with difficult anatomy, stenosis with severe calcifications, stenosis in small vessels (<2.5mm)); and left ventricular wall >8mm detected by transthoracic echocardiography (TTE) in the treatment area.

Exclusion criteria were as follows: women in fertile age, type 1 diabetes or severe complicated type 2 diabetes (diabetic retinopathy or nephropathy), clinically significant anemia (hemoglobin count <120mg/l in males, <110mg/l in females), leukopenia (b-leukocyte count <3.0x10^9^/l), leukocytosis (b-leukocyte count >12.0x10^9^/l), thrombocytopenia (b-thrombocyte count <100x10^9^/l), renal insufficiency (p-creatinine >160mg/l), liver insufficiency (alanine aminotransferase and alcaline phosphatase >2 x normal value), haematuria of unknown origin, severe hypertension (systolic blood pressure >200mmHg or diastolic blood pressure >110mmHg), significant hypotension (systolic blood pressure <90mmHg), significant obesity (BMI >35), acute infection, immunosuppressive medication and significant impairment of the left ventricular function (EF <25% in TTE).

**NOGA mapping and gene transfer**

Patients were admitted to the hospital ward one day prior to the gene transfer procedure. Endocardial gene transfer was performed in the cardiac catheter laboratory. Transseptal puncture was performed via right femoral vein using a transseptal needle and an 8.5Fr steerable introducer catheter (Agilis^TM^ NxT St Jude Medical, St.Paul, Minnesota, USA) under fluoroscopic guidance. After the transseptal puncture unfractionated heparin was given to maintain ACT between 300-350 s. An electroanatomical mapping and injection catheter (NOGA©, Cordis Corp., Johnson & Johnson company, Miami Lakes, USA) was introduced into the left ventricle via the transseptal catheter. For the selection of optimal sites for the gene injections, left ventricular endocardium was mapped spot-by-spot to detect the areas of viable myocardium with reduced contraction (Figure 1). In addition, the baseline positron emission tomography (PET) radiowater imaging (see below) was used to identify myocardial segments with impaired perfusion reserve (MPR) during adenosine-induced stress at baseline.

After mapping the randomization code was opened in the Hospital Pharmacy. The NOGA© catheter was used to inject AdVEGF-D^∆N∆C^ to ten different sites in the hibernating myocardium (Figures 1, 3). The depth of the injections was 5-6 mm and the injections were given 5 to 10 mm apart from each other (200 μl per injection). The control group underwent the same mapping procedure of the left ventricle. Ten sites for gene injections were mapped. However, instead of intramyocardial injections, ten 200 μl injections of 0.9% NaCl solution were injected into the selected sites but with the injection needle withdrawn (i.e. NaCl was injected into the left ventricular cavity). Only the operator performing the injections and Hospital Pharmacy were open to the randomization code. The patients and other personnel were blinded for the study drug throughout the study.

After the gene transfer procedure the patients were monitored at the Cardiac Care Unit until the first postoperative day and if no signs of adverse reactions were detected, they were transferred to a cardiology bed ward. The patients were discharged on the second postoperative day.

**Adenoviral vector and VEGF-D^∆N∆C^**

Replication-deficient E1-E3-deleted serotype 5 adenoviruses were produced in 293 cells using GMP production methods by FinVector Therapies Oy (Kuopio, Finland). Details of the production methods are described elsewhere^1^. AdVEGF-D^∆N∆C^ is an angiogenic and lymphangiogenic growth factor which contains a VEGF homology domain but lacks the N- and C-terminal propeptides^2,3^. Details of the biological effects and signaling of AdVEGF-D^∆N∆C^ have been described elsewhere^4-6^.

**Primary endpoint**

The primary end point of the study was to evaluate safety and feasibility of the intramyocardial AdVEGF-D^∆N∆C^ gene transfer, as measured by acute and late adverse events, laboratory safety parameters, anti-adenovirus antibodies and VEGF-D-levels before the gene transfer and at several time points after the gene transfer.

**Secondary endpoints**

The secondary endpoint was the effect of gene transfer on myocardial perfusion as measured by quantitative ^15^O-water perfusion-PET at baseline and 3 and 12 months after the gene transfer. Other exploratory secondary endpoints were left ventricular function measured by TTE, arrhythmias recorded with 24-hours Holter-recording and values of laboratory parameters. Subjective improvement in symptoms (CCS class), quality of life (QoL) at 3 and 12 months, the need for medication and hospital admissions were also evaluated.

**Laboratory analyses and vital signs**

Blood samples were taken for laboratory tests at baseline, and 1, 6 and 14 days as well as 3, 6 and 12 months after the gene transfer. Enzyme-linked immunosorbent assay was used to measure plasma VEGF-D levels according to manufacturer's instructions (Quantikine DVED00; R&D Systems, Minneapolis). For the analysis of anti-VEGF-D antibodies, 96-well plates were coated with recombinant VEGF-D^∆N∆C^ protein overnight. Dilution series of samples and control antibodies (MAb286, R&D Systems) were incubated on the plates for 2 h and the amount of bound antibodies measured using HRP-conjugated secondary antibodies (A8792, Sigma Aldrich) using TMB detection (T0440 and S5814, Sigma Aldrich). Anti-adenovirus antibodies^7^ and Lp(a) levels^8^ were measured as previously described.

ECG, vital signs, including blood pressure, heart rate and body temperature, were measured at baseline, during and 4 h after the procedure and thereafter daily until discharge, as well as at every follow-up visit.

**Transthoracal echocardiography**

TTE was performed to all patients at baseline. 2-D images with apical, horizontal and vertical projections were used. Left ventricular function was calculated by using Teicholtz-method. After the gene transfer, repeated TTEs were performed daily until discharge for the evaluation of possible pericardial effusions or other complications. TTE was repeated at the 3 and 12 months follow-up visits.

**Myocardial perfusion imaging**

Patients underwent assessment of quantitative myocardial perfusion with PET imaging (GE Discovery, General Electric Medical Systems, Waukesha, WI, USA) at baseline, as well as 3 and 12 months after the gene transfer using ^15^O-H_2_O as described^9^. A dynamic PET scan of the heart was initially performed after injection of ^15^O-H_2_O (900–1100 MBq) as an intravenous bolus over 15s at rest. After decay of radioactivity, a second scan was performed during adenosine-induced stress.

The perfusion studies were analysed using a validated software (Carimas version 2.5, www.turkupetcentre.net/carimasturku) blinded to the treatment, clinical data, time point, and physiologic status^10^. For all imaging analyses, the left ventricle was divided into 17 segments. Regional myocardial blood flow (MBF) of all 17 segments was calculated as an average of three repeated analyses to minimize operator-dependent variability, using a standard anatomic template and a Carimas software. MPR was calculated for each segment as the ratio of MBF during adenosine stress and at rest. To assess changes in MBF and MPR after therapy, two areas of interest were defined: (i) low MPR defined as the myocardial area with the lowest perfusion reserve (i.e. the treatment area with viable, hibernating myocardium in the combined PET and NOGA mapping), and (ii) reference MPR as the myocardial segment with the highest MPR at baseline PET imaging^10^.

**Quality of Life (QoL)**

Health related QoL was assessed with 15 dimensions (15D) questionnaire at baseline and 3 months after the therapy. 15D is a generic, fifteen-dimensional, standardized, self-administered instrument that can be used both as a profile and a single index score measure of QoL^11^. The single index (15D score) on a 0–1 scale, represents overall QoL. The maximum score is 1 (no problems on any dimensions) and the minimum is 0. A chance of ≥0.015 in the 15D score has been shown to be clinically significant^12^. CCS class was evaluated at baseline and during the follow-up using routine methods.

**Statistical analysis**

No outliers were detected or omitted from the analyses. One patient originally referred to the study did not sign informed consent and was not randomized and included in the study. The primary model parameter to analyze repeated measurements was a linear mixed effect model and post-hoc analyses were performed by the least significance method. The differences between the study groups were calculated using the nonparametric Mann-Whitney U-test. Fisher's exact test was used to calculate dichotomous variables. Data is expressed as means ±SD for continuous variables and as absolute and relative frequencies for categorical variables. Odds ratios and 95% confidence intervals were used to evaluate associations. Results were considered statistically significant at a value of p<0.05. Statistical analyses were performed using SPSS Statistics version 21.0.

**References**

1. Westphal M, Ylä-Herttuala S, Martin J, Warnke P, Menei P, Eckland D, Kinley J, Kay R, Ram Z and ASPECT Study Group. Adenovirus-mediated gene therapy with sitimagene ceradenovec followed by intravenous ganciclovir for patients with operable high-grade glioma (ASPECT): a randomised, open-label, phase 3 trial. *Lancet Oncol* 2013;**14**:823-833.
2. Nieminen T, Toivanen PI, Rintanen N, Heikura T, Jauhiainen S, Airenne KJ, Alitalo K, Marjomäki V, Ylä-Herttuala S. The impact of the receptor binding profiles of the vascular endothelial growth factors on their angiogenic features. *Biochim Biophys Acta* 2014;**1840**:454-63.
3. Toivanen PI, Nieminen T, Viitanen L, Alitalo A, Roschier M, Jauhiainen S, Markkanen JE, Laitinen OH, Airenne TT, Salminen TA, Johnson MS, Airenne KJ, Ylä-Herttuala S. Novel vascular endothelial growth factor D variants with increased biological activity. *J Biol Chem* 2009;**284**:16037-48.
4. Jauhiainen S, Häkkinen SK, Toivanen PI, Heinonen SE, Jyrkkänen HK, Kansanen E, Leinonen H, Levonen AL, Ylä-Herttuala S. Vascular endothelial growth factor (VEGF)-D stimulates VEGF-A, stanniocalcin-1, and neuropilin-2 and has potent angiogenic effects. *Arterioscler Thromb Vasc Biol* 2011;**31**:1617-1624.
5. Rissanen TT, Markkanen JE, Gruchala M, Heikura T, Puranen A, Kettunen MI, Kholová I, Kauppinen RA, Achen MG, Stacker SA, Alitalo K, Ylä-Herttuala S. VEGF-D is the strongest angiogenic and lymphangiogenic effector among VEGFs delivered into skeletal muscle via adenoviruses. *Circ Res* 2003:**92**:1098-106.
6. Jia H, Bagherzadeh A, Bicknell R, Duchen MR, Liu D, Zachary I. Vascular endothelial growth factor (VEGF)-D and VEGF-A differentially regulate KDR-mediated signaling and biological function in vascular endothelial cells. *J Biol Chem* 2004;**279**:36148-57.
7. Hedman M, Hartikainen J, Syvänne M, Stjernvall J, Hedman A, Kivelä A, Vanninen E, Mussalo H, Kauppila E, Simula S, Närvänen O, Rantala A, Peuhkurinen K, Nieminen MS, Laakso M, Ylä-Herttuala S. Safety and feasibility of catheter-based local intracoronary vascular endothelial growth factor gene transfer in the prevention of postangioplasty and in-stent restenosis and in the treatment of chronic myocardial ischemia: phase II results of the Kuopio Angiogenesis Trial (KAT). *Circulation* 2003;**107**:2677-83.
8. Tsimikas S, Lau HK, Han KR, Shortal B, Miller ER, Segev A, Curtiss LK, Witztum JL, Strauss BH. Percutaneous coronary intervention results in acute increases in oxidized phospholipids and lipoprotein(a): Short-term and long-term immunologic responses to oxidized low-density lipoprotein. *Circulation* 2004;**109**:3164-3170.
9. Harms HJ, Nesterov SV, Han C, Danad I, Leonora R, Raijmakers PG, Lammertsma AA, Knuuti J, Knaapen P. Clinical value of absolute quantification of myocardial perfusion with 15O-water in coronary artery disease. *Circ Cardiovasc Imaging* 2011;**4**:678-684.
10. Nesterov SV, Han C, Mäki M, Kajander S, Naum AG, Helenius H, Lisinen I, Ukkonen H, Pietilä M, Joutsiniemi E, Knuuti J. Myocardial perfusion quantitation with 15O-labelled water PET: high reproducibility of the new cardiac analysis software (Carimas). *Eur J Nucl Med Mol Imaging* 2009;**36**:1594-602.
11. Sintonen H. The 15D instrument of health-related quality of life: properties and applications. *Annals of Medicine* 2011;**33**:328-336.
12. Alanne S, Roine RP, Räsänen P, Vainiola T, Sintonen H. Estimating the minimum important change in the 15D scores. *Qual Life Res* 2015;**24**:599-606.
