# Supplementary material for: Adenoviral intramyocardial VEGF-DΔNΔC gene transfer increases myocardial perfusion reserve in refractory angina patients: a phase I/IIa study with 1-year follow-up
Source: Eur Heart J. 2017 Jul 31;38(33):2547–55. doi: 10.1093/eurheartj/ehx352 (PMC5837555; doi:10.1093/eurheartj/ehx352)
Supplement: Supplementary Table 1 [file supptab1_val_ehx352.docx]

Supplementary Table 1. Follow-up measurements. P-values for group and baseline comparisons.

|  | **Baseline**  Control AdVEGF-D  n=6 n=24  Control AdVEGF-D | | **1 day**  Control AdVEGF-D  n=6 n=24 | | **6 days**  Control AdVEGF-D  n=6 n=24 | | **14 days**  Control AdVEGF-D  n=6 n=23 | | **3 months**  Control AdVEGF-D  n=6 n=23 | | **12 months**  Control AdVEGF-D  n=6 n=21 | | **p1** |
| --- | --- | --- | --- | --- | --- | --- | --- | --- | --- | --- | --- | --- | --- |
| B-Hb  (g/l) | 143±15  p2=0.820 | 141±16  p2=0.820 | 138±14  p2=0.631  p3=1.000 | 136±16  p2=0.631  p3=0.011 | 137±19  p2=0.935  p3=1.000 | 138±14  p2=0.935  p3 = 0.036 | 135±18  p2=0.618  p3=0.262 | 137±15  p2=0.618  p3=0.053 | 142±17  p2=0.813  p3=1.000 | 141±15  p2=0.813  p3=1.000 | 145±18  p2=0.887  p3=1.000 | 142±18  p2=0.887  p3=1.000 | 0.011 |
| B-Leuc (x10^9^/l) | 8.4±2.6  p2=0.082 | 6.6±1.4  p2=0.082 | 7.8±1.7  p2=0.432  p3=1.000 | 7.0±1.5  p2=0.432  p3=1.000 | 7.2±1.5  p2=0.849  p3=0.653 | 7.6±4.0  p2=0.849  p3=0.290 | 6.9±1.2  p2=0.127  p3=0.236 | 6.1±1.1  p2=0.127  p3=1.000 | 7.9±2.1  p2=0.278  p3=1.000 | 6.9±1.2  p2=0.278  p3=1.000 | 8.6±2.8  p2=0.140  p3=1.000 | 7.0±1.2  p2=0.140  p3=1.000 | 0.569 |
| B-Thromb (x10^9^/l) | 274±70  p2=0.082 | 214±63  p2=0.082 | 247±53  p2 = 0.038  p3=1.000 | 186±61  p2 = 0.038  p3 = 0.001 | 289±38  p2=0.039  p3=1.000 | 218±75  p2=0.039  p3=1.000 | 269±29  p2=0.114  p3=1.000 | 229±72  p2=0.114  p3=0.240 | 247±61  p2=0.090  p3=1.000 | 201±59  p2=0.090  p3=1.000 | 261±58  p2=0.075  p3=1.000 | 205±86  p2=0.075  p3=1.000 | 0.001 |
| P-Alt  (U/l) | 34±17  p2=0.347 | 32±28  p2=0.347 | NA | NA | 39±20  p2=0.174  p3=1.000 | 30±17  p2=0.174  p3=1.000 | 31±13  p2=0.604  p3=1.000 | 30±15  p2=0.604  p3=1.000 | 32±24  p2=0.937  p3=1.000 | 29±15  p2=0.937  p3=1.000 | 34±16  p2=0.316  p3=1.000 | 26±11  p2=0.316  p3=0.374 | 0.277 |
| P-CRP  (mg/l) | 4±2  p2=0.860 | 4±2  p2=0.860 | 6±3  p2=0.860  p3=1.000 | 9±8  p2=0.860  p3=0.854 | 5±3  p2=0.940  p3=1.000 | 13±29  p2=0.940  p3 = 0.022 | 3±0  p2=0.643  p3=1.000 | 4±3  p2=0.643  p3=1.000 | 4±2  p2=0.232  p3=1.000 | 3±0  p2=0.232  p3=1.000 | 5±3  p2=0.289  p3=1.000 | 3±0  p2=0.289  p3=1.000 | 0.022 |
| P-Tnt  (ng/l) | 10±4  p2=0.233 | 14±6  p2=0.233 | 95±56  p2=0.897  p3 = 0.004 | 93±39  p2=0.897  p3= 0.001 | 12±2  p2=0.237  p3=1.000 | 21±17  p2=0.237  p3=1.000 | 11±5  p2=0.211  p3=1.000 | 16±8  p2=0.211  p3=1.000 | 14±4  p2=0.914  p3=1.000 | 16±9  p2=0.914  p3=1.000 | 14±6  p2=0.836  p3=1.000 | 14±9  p2=0.836  p3=1.000 | 0.001 |
| Systolic BP (mmHg) | 158±16  p2=0.065 | 141±20  p2=0.065 | 134±25  p2=0.896  p3=0.168 | 131±16  p2=0.896  p3=0.314 | NA | NA | NA | NA | 151±21  p2=0.328  p3=1.000 | 141±22  p2=0.328  p3=1.000 | 148±21  p2=0.408  p3=1.000 | 140±20  p2=0.408  p3=1.000 | 0.886 |
| Diastolic BP (mmHg) | 88±10  p2=0.210 | 80±13  p2=0.210 | 74±12  p2=0.694  p3=0.147 | 71±15  p2=0.694  p3= 0.018 | NA | NA | NA | NA | 83±11  p2=0.192  p3=1.000 | 76±13  p2=0.192  p3=1.000 | 82±21  p2=0.629  p3=1.000 | 76±13  p2=0.629  p3=1.000 | 0.018 |
| CCS class | 2.67±0.52  p2=0.561 | 2.83±0.38  p2=0.561 | NA | NA | NA | NA | NA | NA | 2.17±0.75  p2=0.477  p3=1.000 | 2.43±0.59  p2=0.477  p3=1.000 | 2.00±0.71  p2=0.801  p3=0.230 | 2.11±0.47  p2=0.801  p3=0.001 | 0.001 |

Mean±SD. Significances: p1 = p-value for interaction time x group; p2 = significance of group difference in the given time point; p3 = significance of difference between given time point and baseline. Abbreviations: Hb = Hemoglobin, Alt = alanine amino transferase, CRP = C-reactive protein, TnT = Troponine-T, BNP = N-terminal pro-brain natriuretic peptide, BP = blood pressure. CCS = Canadian Cardiovascular Society class.
