# Supplementary material for: Adenoviral intramyocardial VEGF-DΔNΔC gene transfer increases myocardial perfusion reserve in refractory angina patients: a phase I/IIa study with 1-year follow-up
Source: Eur Heart J. 2017 Jul 31;38(33):2547–55. doi: 10.1093/eurheartj/ehx352 (PMC5837555; doi:10.1093/eurheartj/ehx352)
Supplement: Supplementary Table 2 [file suppltab2_val_ehx352.docx]

Supplementary Table 2. Myocardial perfusion reserve and Lp(a) levels of the AdVEGF-D^dNdC^ treated patients.

P-values for group and baseline comparisons.

|  | **Baseline** | | | **3 months** | | | **12 months** | | | **p1** |
| --- | --- | --- | --- | --- | --- | --- | --- | --- | --- | --- |
| Lp (a) Tertiles | 3rd  n=5 | 2nd  n=5 | 1st  n=5 | 3rd  n=5 | 2nd  n=5 | 1st  n=4 | 3rd  n=5 | 2nd  n=5 | 1st  n=5 |  |
| Lp(a) mg/dl | 52.7±13.3  p2 =0.002 | 11.8±6.4  p2=0.002 | 3.8±2.0  p2=0.002 | 53.9±20.5  p2=0.001  p3=1.000 | 11.0±5.0  p2=0.001  p3=1.000 | 3.2±1.3  p2=0.001  p3=0.944 | 59.1±19.6  p2=0.001  p3=0.447 | 13.8±4.1  p2=0.001  p3=0.408 | 3.3±0.7 p2=0.001  p3=1.000 | 0.203 |
| Treated area MPR | 0.94±0.32  p2=0.733 | 1.16±0.45  p2=0.733 | 1.03±0.34  p2=0.733 | 1.43±0.45  p2=0.650  p3=0.082 | 1.39±0.57  p2=0.650  p3=0.647 | 1.17±0.38  p2=0.650  p3=0.163 | 1.76±0.41  p2=0.139  p3 = 0.023 | 1.42±0.53  p2=0.139  p3=0.546 | 1.22±0.43 p2=0.139  p3=0.324 | 0.089 |

Mean±SD. Tertiles refer to Lp(a) at baseline. Significances: p1 = p-value for interaction time x tertiles; p2=group difference at the given time point; p3=difference between time point and baseline. MPR=myocardial perfusion reserve.
